# Plasmonically enhanced metal–insulator multistacked photodetectors with separate absorption and collection junctions for near-infrared applications

**DOI:** 10.1038/srep42349

**Published:** 2017-02-09

**Authors:** Sina Abedini Dereshgi, Zulkarneyn Sisman, Kagan Topalli, Ali Kemal Okyay

**Affiliations:** 1 Department of Electrical and Electronics Engineering, Bilkent University, Ankara 06800, Turkey; 2 UNAM-National Nanotechnology Research Center, Bilkent University, Ankara 06800, Turkey; 3 Institute of Material Science and Nanotechnology, Bilkent University, Ankara 06800, Turkey

## Abstract

Plasmonically enhanced metal-insulator-metal (MIM) type structures are popular among perfect absorbers and photodetectors in which the field enhancement (for increased absorption) mechanism is directly coupled with collection (photocurrent) processes. In this work we propose a device structure that decouples absorption and collection parts for independent optimization. Double-stacked MIM (i.e. MIMIM) photodetectors operating in the near-infrared (NIR) spectrum up to 1200 nm wavelength are demonstrated. In the absorbing MIM (at the top side), we have used Silver nanoparticles resulting from dewetting, yielding a very low reflection of 10% for the most part of the 400 to 1000 nm wavelength range. An unconventional plasmonic material, Chromium, exhibits an absorption peak of over 80% at 1000 nm. The complete device has been fabricated and the photo-collection tunneling MIM (at the bottom) suppresses the leakage current by metal workfunction difference. An optimized stack consisting of Silver – Hafnium Oxide – Chromium – Aluminum Oxide – Silver nanoparticles (from bottom to top) yields a dark current of 7 nA and a photoresponsivity peak of 0.962 mA/W at 1000 nm and a full width at half maximum of 300 nm, while applied bias is 50 mV and device areas are 300 μm × 600 μm.

With the advent of plasmonics in the recent decades there has been a lot of work ranging from exotic geometries as absorbers to photovoltaic applications. The concurring trend of scaling down the electronic and photonic devices has led to thriving devices that target this requirement by employing plasmonic nanoscale active layers. While Otto and Kretchmann configurations were the presumptive methods to excite surface plasmons (SP), gratings^1^,^2^,^3^, nanoparticles^4^,^5^,^6^,^7^,^8^ and nanopillars^9^,^10^,^11^ made way to more efficient, omnidirectional and compact structures thanks to advanced patterning methods such as electron beam lithography. MIM structures attract interest due to their ability to efficiently trap the incident light within their spacer insulators^1^,^3^. Owing to suitable plasma frequency of Silver metal, grating structures made of Silver have been investigated to enhance absorption in Silicon for solar cell applications^12^. Fang *et al*. report using Silver hole-array as corrugated top layer in an MIM configuration in the visible spectrum^13^. A more exotic trapezoid array was reported that manifests over 50 percent absorption within the whole visible spectrum^14^. There are also reports of using MIM structures for efficient infrared absorbers^15^,^16^. A combination of multi-harmonic geometries is also reported in the literature to combine the absorption peaks resulting in broadband MIM absorbers^17^.

While it is plausible to fabricate perfect absorbers using patterning techniques such as electron beam lithography, prohibitive costs associated with such methods cast doubt on their practical use for large volume manufacturing. Therefore, dewetting of thin metallic films has emerged as a strong candidate to form nanostructures in a low cost and large-area compatible fashion. Not only does dewetting result in low-cost fabrication of active plasmonic layers, but also the structures formed with this technique exhibit broad absorption spectra. However, there is a trade-off between the spectral bandwidth and the peak height of the absorption profile. Dewetting approach has also been utilized for solar cells to increase scattered light reaching the active region^18^,^19^.

Incident light on nanoparticles excites localized surface plasmons (LSPs) or surface plasmons (SPs) and their non-radiative decay results in energetic hot electron-hole pairs that can be harnessed as photocurrent at a Schottky junction^20^,^21^. In case of traditional MIM type absorber designs, the burden of both absorption and photocurrent generation lies on the same junction which places stringent restrictions on device engineering to comply with desirable electrical and photonic characteristics simultaneously. Besides, Kretchmann configuration is needed to avoid competing photocurrents resulting from LSP excitation only^22^. Another configuration for MIM is a design with one metal layer comprised of nanoparticles. In such a structure, we need a transparent conductive oxide (TCO) that encapsulates nanoparticle layer to collect the current. This system would be inefficient due to the fact that we have two counter acting photocurrents, one from LSP excitation in nanoparticle layer and the other from SP excitation in the bottom metal which is therefore not reported in literature to the best of our knowledge. In order to remove restrictions of MIM, a new paradigm is proposed to decouple absorbing junction from the photocollection junction allowing the independent optimization of optical and electrical processes. This can be achieved in an MIMIM type multi-stack structure^23^.

Traditionally Silver and Gold are metals of choice to construct plasmonic nanostructures in the visible^24^ and NIR regions^25^. In this work, we also introduce non-conventional metals to be used in different layers of MIMIM multi-stack, especially the absorber layer in NIR region. The top metal is desired to be a good scatterer with low loss while the absorber metal (in the middle) is desired to have a high loss. Materials with the highest absorption coefficients may not be the most suitable metals for this work since the field penetration into the material is also crucial for significant absorption to take place. Despite their high absorption coefficient (*k*), widely used plasmonic metals are typically good reflectors due to high optical refractive indices (*n*′). Electric field does not efficiently penetrate into materials with high optical refractive index. Surface plasmon polariton (SPP) quality factor is a suitable metric for the attenuation of fields in a material, taking into consideration the absorption coefficient as well as the permittivity (*ε* = *n*^*2*^). In this paper, first absorbing top MIM is investigated computationally and experimentally and afterwards, the photocollection MIM is optimized and the overall photoresponsivity performance is examined. Figure 1(a) illustrates the schematic of the MIMIM structure as well as contact metals in detail. The distribution of particle sizes is depicted in Fig. 1(c) which demonstrates an average size of 97.11 nm, average distance of 161.4 nm between particles and an average height of 50 nm for particles.

## Results

### Absorption (MIM)

Simulations the MIMIM structure using FDTD Solutions (by Lumerical) show that the absorption peak lies in the NIR range of interest for insulator (Al2O3) thickness values between 30–50 nm. The thickness of the insulator is chosen and fixed at 40 nm for all the simulations and experiments. We use atomic layer deposition system (ALD) to deposit insulators and control their thicknesses precisely. The main reason for choosing Aluminum Oxide as the spacer is the fact that among dielectrics, Aluminum Oxide is the least defective one and possesses the lowest k value (imaginary part of refractive index) compared to others like Zinc Oxide, Titanium Oxide and Hafnium Oxide.

**Figure 1:**
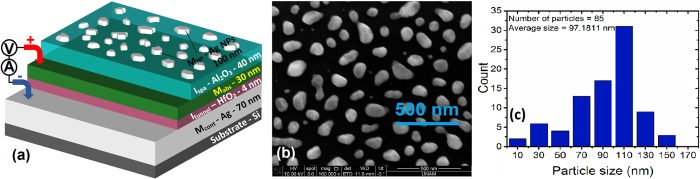
(**a**) Schematic of the MIMIM device, comprised of metal nanoparticles (M_NP_), spacer insulator (I_spa_), absorbing metal (M_abs_), tunneling insulator (I_tunnel_) and bottom contact metal (M_cont_), (**b**) SEM image of the nanoparticles after annealing 10 nm Silver film for 20 min at 500 °C and (**c**) the size distribution of particles from SEM image of part (**b**).

Ultra-thin, metal films like gold (<10 nm) are not stable due to surface forces at metal-air interface. As the metal film thickness is a few nanometers, not only the deposited film is unstable but also it can barely be regarded as a continuous film. When the thicknesses are in the order of 10 nm, the film is in a thermodynamically metastable state meaning that under some excitation, the film will evolve by agglomerating into islands to minimize the surface area with air^26^. The breaking of the continuous film into islands by such a process is called dewetting. The size and morphology of the resulting nanoclusters (islands) on the surface highly depend on the layer beneath metal, the metal type and excitation parameters. 10 nm thick Silver was deposited on Aluminum Oxide film via thermal evaporation, and a consecutive rapid thermal annealing (RTA) at 500 °C for 20 min was carried out. The scanning electron microscope (SEM) image of the resulting nanoparticles is given in Fig. 1(b), which shows nanoparticles are approximately on the order of 100 nm laterally^27^.

**Figure 2:**
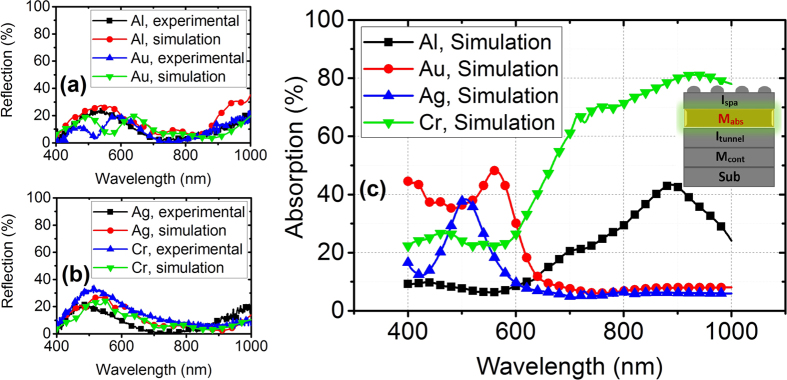
Measured and computational 3D simulation results of reflection from a surface of MIMIM (Ag nanoparticles - Al_2_O_3_ - M_abs_ - HfO_2_ – Ag bottom contact) with M_abs_ chosen to be (**a**) Aluminum and Gold, (**b**) Silver and Chromium, (**c**) simulated absorption percentage in different absorbing metals. Inset of (**c**) illustrates the layer being studied (i.e. absorbing metal).

**Figure 3:**
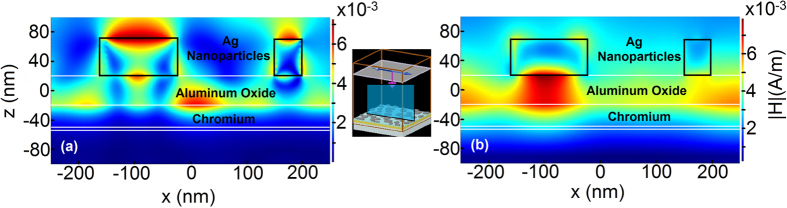
Computed H-field distribution at wavelengths of (**a**) 400 nm and (**b**) 1000 nm for MIMIM structure with Chromium absorbing (middle) metal, at a cross section of sample which includes and bisects two nanoparticles. The inset between two figures illustrates the cross section plane.

The simulated MIMIM structures are shown in Fig. 1(a). The resulting normal reflection measurements and simulations for four different absorbing metals (Aluminum, Gold, Silver and Chromium) are depicted in Fig. 2(a) and (b). Figure 2(c) shows the amount of absorption in the absorbing metal (M_abs_). Both simulation and experimental results are normalized to Aluminum coated mirrors. The simulations are carried out in 3D while the nanoparticles are extracted from the SEM image into the simulation environment assuming a 50 nm thickness which is the only simplification in simulation (inset of Fig. 3). The 50 nm average thickness is calculated from the comparison between volume of deposited film and area of resulting particles after dewetting. The *n, k* data for Aluminum Oxide is extracted using J.A. Woollam Co. Inc. VASE. The mesh size is 3 nm in z-direction (perpendicular to the sample surface) and 15 nm in x and y directions and the simulation area is 500 nm × 500 nm.

Spectral reflection results calculated computationally agree well with experimental ones. The little discrepancy between simulation and experiment stems from the fact that we have approximated the thickness of particles to be 50 nm for all of them. Moreover, there is generally a blue-shift in experimental reflection results compared to the simulation results which is attributed to the tarnishing of silver. This is justified once more in the photoresponsivity results in the following sections. It can be deduced from Fig. 2(c) that Aluminum and Chromium show loss and absorption peaks of 42 percent and 81 percent around 800 nm and 1000 nm, respectively while Silver and Gold are not desirable since they yield very low absorption beyond 600 nm. The observed broad absorption peaks are innate properties of random nanoparticle enhanced absorbers. The absorption of Chromium based structure is highly broadband and extends further into NIR spectrum. Investigating the field profile (Fig. 3) can be insightful in understanding field penetration. The field profiles in the H-field simulation of all of the absorbing metals are analogous with small differences; thus, the results for Chromium is presented here. It is deduced that field penetration from Chromium to bottom layers is negligible.

**Figure 4:**
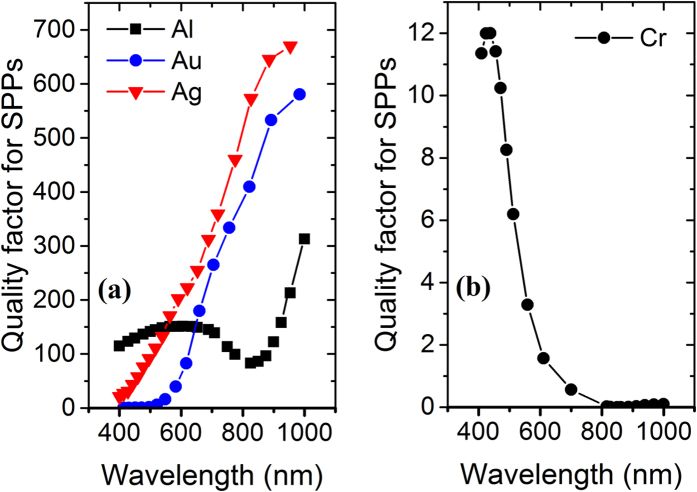
SPP quality factor for (**a**) Aluminum, Gold, Silver and (**b**) Chromium.

At 400 nm (Fig. 3(a)) we observe higher order plasmonic modes with confined field mostly within Silver nanoparticle – air interface which results in excitation of LSPs and absorption mainly occurs in Silver nanoparticles. At 1000 nm (Fig. 3(b)), however, the field is confined within the Aluminum Oxide – absorbing metal interface giving rise to absorption probability through excitation of SPs in the metal. For Silver and Gold absorbing metals, however, the light is reflected and confined in nanoparticle region mainly. The absorption in Aluminum Oxide is negligible^27^. One can assume that the same H-field profile may lead same absorption in all absorbing metals. To confront this, we propose scrutinizing electric SPP quality factor of metals. Figure 4 illustrates SPP quality factor for four different metals using their respective permittivity data, taking Palik model for Aluminum and Chromium^28^, Johnson and Christy for Gold^29^ and CRC for Silver^30^.

SPP quality factor *Q*_*SPP*_ is defined as


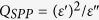


where ε′ and ε″ are the real and imaginary part of permittivity respectively^31^. While there is little attention dragged to choosing the absorbing or scattering metals from the existing ones, this concept fits best for such a purpose. A metal is considered a good absorber in a wavelength of interest, when it has low real permittivity (to boost field penetration) and high complex permittivity (high absorption). Therefore, for this purpose Aluminum and specifically Chromium show minima of SPP quality factor and are the most appropriate choices for the target wavelength range. There is a relative minimum for Aluminum which accounts for increased absorption. The situation is pronounced for Chromium because its plasma frequency occurs at 850 nm and for NIR absorption, it proves to be most suitable. It is worth pointing out that using Silver for nanoparticles is also the best choice due to its high SPP quality factor within 400 nm to 1000 nm wavelengths which justifies high scattering after a preceding excitation of LSP^27^.

### Photodetector Design (MIMIM)

In order to collect the resulting hot electrons or holes, a tunneling design is proposed. There is another possible choice which is using a semiconductor directly in Schottky contact with absorbing metal. The latter is rather inefficient since the dark current typically is rather high in Schottky contacts compared to the tunneling counterparts. Out of the four fabricated samples that are investigated, two are Aluminum – Hafnium Oxide – Aluminum or Chromium absorbing metal – Aluminum Oxide – Silver nanoparticles (from bottom to top), and the other two are the same structures without Silver nanoparticle layer (MIMI) as references to the first two, respectively. Studying these samples emphasizes plasmonically enhanced absorption as well as the effect of the choice of absorbing metal type. One important design consideration for the junction is the substantial role of absorbing metal thickness in photocurrent. If the absorbing metal is chosen to be too thick, ohmic loss would degrade photoresponsivity. Therefore, the thickness of absorbing metals is chosen to be 30 nm. The reason for choosing 30 nm thick top metals is that the skin depths of metals in the wavelengths of interest are approximately 20 nm^32^ and slightly thicker metal layers are chosen in order to block any possible penetration of light through the stack to bottom metal which would result in counteracting reverse photocurrent. This counteracting photocurrent would be as a result of hot-hole generation in bottom metal where a negative bias already exists.

In order to design an efficient tunneling junction, the main parameters are the choice of bottom metal and tunneling insulator. The available and suitable insulators for tunneling purpose in our ALD system are Aluminum Oxide and Hafnium Oxide. Aluminum Oxide is chemically etched during lithography developing steps in the process (see methods). Given the fact that the tunneling insulator is thin, we chose Hafnium Oxide which is also a high-k dielectric. The thickness of the insulator is critical in performance. For thicknesses above 4 nm the photocurrent is highly suppressed due to the exponential dependence of tunneling current on insulator thickness. For thicknesses lower than 4 nm on the other hand, we observe poor uniformity of insulator film. On the grounds of this discussion the tunneling insulator was selected to be 4 nm.

**Figure 5:**
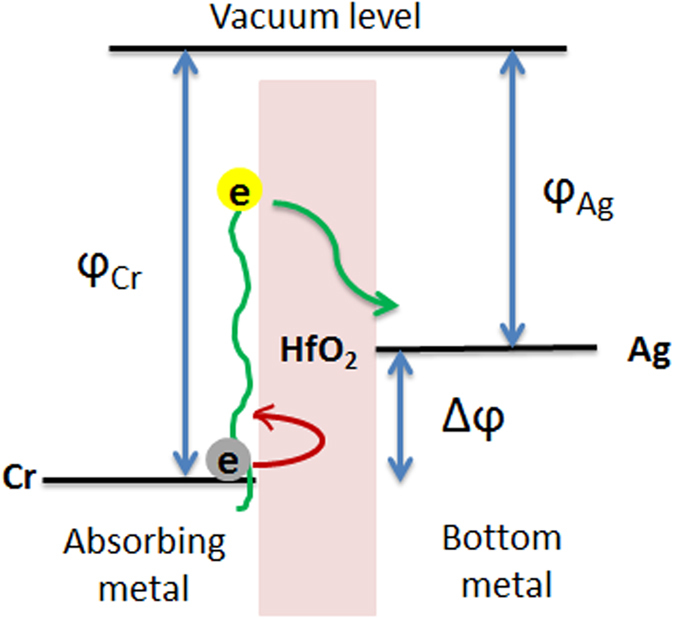
Energy band diagram of the photocollecting tunneling junction with Chromium and Silver contacts, after non-radiative decay of SPs. Green arrows illustrate the transport of energetic electrons and the red one shows blocking the flow of the electrons in conduction band of the absorbing metal.

Another design parameter which is the bottom contact metal was chosen to be Silver. The main reason for this choice was to create an offset in the metal work functions at the two sides of the tunneling insulators. This offset facilitates the suppression of dark (leakage) current which is highly preferable in photodetector performance. Of the possible low resistivity choices, Silver revealed the best performance for both devices with Aluminum and Chromium absorbing metals, due to the fact that it has lower work function than the two absorbing metals. We could use a bottom metal with higher work function than that of Aluminum and Chromium for hot-hole collection but the scarcity of such low resistive metals lead us to Silver to collect hot electrons. The energy band diagram schematic of the tunneling junction is illustrated in Fig. 5.

## Discussion

**Figure 6:**
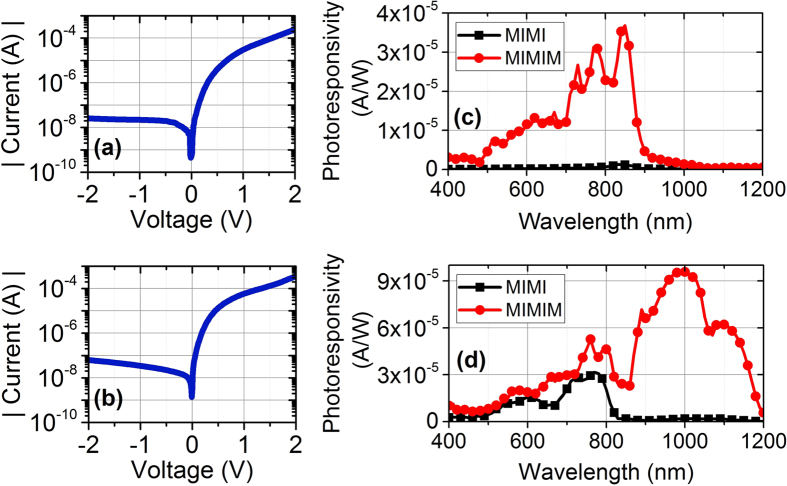
I–V characteristics of MIMIM (Ag nanoparticles - Al_2_O_3_ - absorbing metal - HfO_2_ - Ag bottom contact) devices with (**a**) Aluminunm absorbing metal and (**b**) Chromium absorbing metal, photoresponsivity at applied negative 50 mV bias for MIMI (without Ag nanoparticles) and MIMIM devices respectively with (**c**) Aluminum absorbing metal and (**d**) Chromium absorbing metal.

The applied bias is negative as with the probes illustrated at Fig. 1(a). With such a bias we collect hot electrons and suppress the dark current flow. Since electrons are energetic they can overcome the barrier and work function offset with appropriate applied bias. The dark I-V curves of devices with aluminum and chromium are depicted in Fig. 6(a) and (b) respectively, which justify the work function offset. The device areas are 300 μm × 600 μm. One important and favorable issue is that the I-V curves go through negligible change after dewetting which would not be the case if we used Schottky junctions. In order to discover the most optimum bias, we increase negative bias as long as we observe increase in photocurrent. After an optimum point, which happens to be a negative 50 mV bias, the photocurrent does not increase anymore and dark current increases solely. This phenomenon also solidifies the fact that our measured current is photocurrent. Fig. 6(c) and (d) represent photoresponsivity of MIMIM devices with and without Silver nanoparticles for Aluminum and Chromium respectively, measured at a negative bias of 50 mV.

As it can be inferred from Fig. 6, the observed dark currents are 5 nA and 7 nA for devices with Aluminum and Chromium absorbing metals respectively. The photocurrent results are highly in accordance with the simulated absorptions of Fig. 2(c). Photoresponsivity of MIMIM device with Aluminum has a peak value of 36.84 μA/W at 850 nm which is 29 times the peak value of same device without nanoparticles (the reference device for Aluminum case). The outstanding performance occurs for the MIMIM device with Chromium absorbing metal which exhibits a peak photoresponsivity of 96.24 μA/W at 980 nm which represents 3 times increase compared to the peak value of same device without nanoparticles (the reference device for Chromium case). This is also a 2.6 times increase compared to MIMIM device with Aluminum. The full width at half maximum is more than 300 nm due to the use of random sized nanoparticles. Thus, our best result justifies almost an order of magnitude enhancement compared to MIMI device with Aluminum, which itself has a little absorption peak around 850 nm already. Besides, our Chromium result exhibits more than an order of magnitude enhancement in photoresponsivity over the reported spin-coated MIMIM structures^23^.

It should be pointed out that the photoresponsivity curves follow the simulated absorption curves quite acceptably. There is a red-shift in photoresponsivity results compared to absorption results of Fig. 2(c), which is attributed to the oxidation of Silver nanoparticles. To vindicate this statement, Fig. 2(a) and (b) results can be taken into account. In those results there is a blue-shift in experimental reflection results compared to those of simulations (reflection trend is the opposite of absorption trend) due to oxidation of Silver nanoparticles that is inevitable. Moreover, the smoother photoresponsivity curve of Chromium is because of higher photocurrent for this sample that is far larger than our measurement noise level.

## Conclusion

The demonstration of absorbers and photodetectors were investigated in this work with focus on NIR region. We investigated the effect of four different bottom metals in plasmonically enhanced MIM absorbers with silver nanoparticles. After identifying MIMs with highest absorption in bottom metals of them, which happen to be Aluminum and Chromium, the complete MIMIM photodetector was fabricated in which the energy band engineering is done such that the dark current is suppressed and the photocurrent mechanism is based on tunneling of hot electrons. In an MIMIM structure, light falling on the particles excites SPs in the middle absorbing metal. The non-radiative decay of SPs result in hot EHPs and hot electrons are directed to the bottom metal by tunneling. We point out Chromium as an efficient absorbing metal due to high loss and suitable plasma frequency that happens in NIR region rather than UV for other metals of choice here. The resulting absorption and photocurrent curves are highly in agreement. Our photodetector design manages to decouple absorption and photocollection parts which facilitates design of efficient photodetectors by engineering each layer independently.

## Methods

### Fabrication

A Silicon wafer with unintentional doping was used as the substrate. The substrate was cleaned in Acetone, Isopropanol and de-ionized water for 10 minutes each using ultrasonic agitation. Afterwards, 70 nm silver was deposited with thermal evaporation at a base pressure of 2–4 × 10^−6^ Torr. Afterwards the sample was masked with a small margin (to get the negative contact) and a deposition of hafnium oxide at 200 °C was carried out with Cambridge Nanotech Inc., Savannah S100 ALD system using Trimethylhafnium and milli-Q water as Hafnium and Oxygen precursors respectively with a Nitrogen flow of 20 sccm. For a lift-off process, HMDS and AZ5214 photoresist was spin coated and exposed to UV light using mask aligner and was developed in AZ400K solution, a follow up deposition of 30 nm absorbing metal (either Aluminum or Chromium) was deposited in the same conditions as the negative contact metal and acetone was used for the lift off process. Afterwards, 40 nm Aluminum Oxide was deposited in the same conditions in ALD using Timethylaluminum and milli-Q water as Aluminum and Oxygen precursors respectively. Then, using an optimized inductively coupled plasma (ICP) dry etching recipe^33^, Aluminum Oxide was patterned to form the positive contact. At last, 10 nm thick Silver was deposited on Aluminum Oxide film via thermal evaporation at 2–4 × 10^−6^ Torr, and a consecutive rapid thermal annealing (ATV Technologie GmbH, SRO-704 RTA) step was carried out at 120 sccm N_2_ flow rate. Temperature was ramped up to 500 °C in 5 min, followed by 20 min annealing at constant temperature, 500 °C. At the end of the annealing step, the temperature is passively reduced to 250 °C followed by manually removing the sample to facilitate faster cooling which resulted in some oxidation^27^.

### Characterization

**Figure 7:**
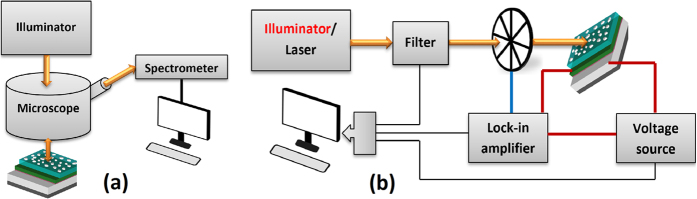
(**a**) Schematic of reflection measurement setup, (**b**) schematic of the spectral photoresponsivity measurement setup.

We used a custom reflection measurement setup comprised of a Halogen illuminator connected to a microscope and directed perpendicular to sample and the reflected light from the microscope was fed to a Newport OSM2 spectrometer and the data was collected by interfacing the spectrometer with PC (Fig. 7 (a)). All measurements were normalized to an Al coated reference sample.

The dark I-V measurements of the devices were conducted using KEITHLEY 2401 Sourcemeter. In order to measure photoresponsivity for the wavelengths between 400 to 800 nm, we used a Xenon-lamp illuminator. The output of the lamp goes through an Oriel 74004 monochromator (1/8 meter, 1200 lines/mm grating). The monochromatic light was collimated and chopped with a mechanical chopper and the chop speed (380 Hz) was read by a lock-in amplifier (SR830). The short circuit current was measured by the lock-in amplifier. Also, a calibrated Si detector was used at the sample position to collect the light source power at each wavelength step. For the wavelengths between 800 to 1300 nm, a similar setup was used, except that the source was a super continuum laser (Fianium) which was monochromated by an acousto-optic transmission filter (AOTF - Crystal Tech.) and the chopper speed was 960 Hz. The spectral power was detected with a calibrated InGaAs detector (Fig. 7(b)).

## Additional Information

**How to cite this article:** Dereshgi, S. A. *et al*. Plasmonically enhanced metal–insulator multistacked photodetectors with separate absorption and collection junctions for near-infrared applications. *Sci. Rep.* **7**, 42349; doi: 10.1038/srep42349 (2017).

**Publisher's note:** Springer Nature remains neutral with regard to jurisdictional claims in published maps and institutional affiliations.
